# A homoharringtonine-based induction regimen for the treatment of elderly patients with acute myeloid leukemia: a single center experience from China

**DOI:** 10.1186/1756-8722-2-32

**Published:** 2009-07-30

**Authors:** Jianmin Wang, Shuqing Lü, Jianmin Yang, Xianmin Song, Li Chen, Chongmei Huang, Jun Hou, Weiping Zhang

**Affiliations:** 1 Department of Hematology, Changhai Hospital, Second Military Medical University, Shanghai, PR China

## Abstract

**Background and purpose:**

The response to remission induction in elderly patients with acute myeloid leukemia (AML) remains poor. The purpose of this paper is to evaluate the efficacy and toxicity of a plant alkaloid, homoharringtonine, in combination with cytarabine as an induction therapy for AML in elderly patients (≥60 years).

**Results:**

Twenty-three patients were treated with the HA regimen consisting of homoharringtonine (2 mg/m^2^/day for 7 days) and cytarabine (Ara-C, 100 mg/m^2^/day for 7 days). The overall response rate was 56.5% with complete remission (CR) rate of 39.1% and partial remission of 17.4%. There was no early death in this cohort of patients. The estimated median overall survival (OS) time of all patients was (12.0 ± 3.0) months. The estimated OS time of the CR patients was 15 months. The estimated one-year OS rate of all patients treated with HA protocol was (49.3 ± 13.5) %. The estimated one-year OS rate of the CR patients was (62.5 ± 17.1) %.

**Conclusion:**

HA is a suitable induction regimen for elderly patients with AML, with relatively low toxicity and reasonable response rate.

## Introduction

The standard induction remission protocol in the treatment of acute myeloid leukemia (AML) in adult patients is 3-day daunorubicin (DNR) and 7-day cytarabine (Ara-C) [1]. Over the 25 years in which this protocol has been in use, a considerable number of strategies have been developed with the goal of improving the efficacy of this protocol, including the substitution of alternative anthracyclines such as idarubicin [2,3]. Although the anthracycline-based protocol has a high overall complete remission (CR) rate in the treatment of AML, the outcome in elderly AML patients remains unsatisfactory. Specifically, even though a high percentage of elderly AML patients, including those with unfavorable prognosis, respond to chemotherapy and survive longer than patients who either refuse treatment or receive supportive treatment alone, many proceed to develop serious and often fatal complications [4,5].

Homoharringtonine (HHT) is a plant alkaloid, derived from the Cephalotuxus fortuneii tree, which for the past 30 years has been used in China for the treatment of AML and chronic myeloid leukemia (CML). HHT and its analogs, such as harringtonine, are inhibitors of protein synthesis whose effects are both dose- and time-dependent. HHT has significant potential synergistic effects with Ara-C. Its cytotoxicity is cell-cycle specific, primarily affecting cells in G1 and G2 phases. HHT is a suitable candidate for the treatment of elderly patients, because it has relatively mild extramedullary toxicity and lacks anthracyclin-like myocardial toxicity [6-12].

We present here a retrospective analysis of the outcome of HHT and Ara-C induction regimen (HA) for the treatment of elderly AML patients admitted to this hospital.

## Patients and methods

### Clinical Data

We treated 23 newly diagnosed elderly (≥ 60 years) non-M3 AML patients in this hematology centre between January 1996 and December 2008 with HHT-based protocol. All patients fulfilled the following criteria: the diagnosis of AML was established according to the standard French-American -British (FAB) cytological and cytochemical criteria; there was no major comorbidity, with normal liver, kidney, heart, and lung function; and informed consent was obtained. All patients underwent full clinical examinations and assessment of blood counts. At the same time, ALT, AST, bilirubin, blood glucose, alkaline phosphatase, and creatinine levels were determined before each course of chemotherapy. Electrocardiograms were performed on all patients before chemotherapy. Patients with karyotypes of t(8;21), inv(16), or t(16;16) were considered to have good-risk cytogenetics; -5, -7, del(5q), del(7q), del(9q), 11q23, abnormal 20q, abnormal 21q, inv(3q), t(6;9), t(9;22), or complex cytogenetics (at least three unrelated cytogenetic abnormalities) were considered to be poor-risk cytogenetics; normal cytogenetics and other miscellaneous single abnormalities were considered to be intermediate-risk cytogenetics.

### Therapeutic Protocols

Each patient received at least one course of induction chemotherapy with HA protocol (HHT 2 mg/m^2 ^daily for 7 days as intravenous (IV) infusion over 4 h; and Ara-C 100 mg/m^2 ^daily for 7 days as continuous IV infusion). Upon recovery of the peripheral blood count, bone marrow (BM) aspiration was performed to assess the response to treatment. For patients who did not achieve CR after the first course of induction therapy, the same induction protocol was repeated once if the decrease of BM blasts was more than 60%. Otherwise, the patients were deemed to be induction failure, and second-line induction therapy was administered per treatment guidelines of this institution.

Granulocyte colony-stimulating factor (5 mcg/kg) was administered subcutaneously daily if the neutrophil count fell below 0.5–1.0 × 10^9^/L after chemotherapy, and terminated when the neutrophil count rose above 1.0–2.0 × 10^9^/L. Blood and platelet transfusion and anti-infection treatment were given according to standard protocols. Consolidation therapy was administered after the achievement of CR as the following: HA regimen as described above, DA (DNR, 30 mg/m^2 ^daily for 3 days, Ara-C 100 mg/m^2 ^daily for 7 days) or IDA (idarubicin, 6 mg/m^2 ^daily for 3 days, Ara-C 100 mg/m^2 ^daily for 7 days), EA (etoposide, 60 mg/m^2 ^daily for 5 days, Ara-C 100 mg/m^2 ^daily for 7 days) and MA (mitoxantrone 6 mg/m^2 ^daily for 3 days, Ara-C 100 mg/m^2 ^daily for 7 days) by turns. In patients older than 70 years, or those who experienced very severe complications, the doses of chemotherapeutic drugs were reduced by 20–50 percent, and/or the duration time of chemotherapy was shortened to 5 days.

### Response criteria

CR was defined as normal hematopoiesis of bone marrow, including neutrophils ≥ 1.5 × 10^9^/L, platelets ≥ 100 × 10^9^/L, blasts in bone marrow ≥ 5%, no blasts in peripheral blood, and the absence of extramedullary disease. PR was defined by bone marrow blasts between 5% and 20% and peripheral blood blasts < 5%, with neutrophils > 1.5 × 10^9^/L and platelets > 50 × 10^9^/L. NR (no response) was assigned to patients who did not fulfill the above criteria. Early toxic death (ED) was defined as death following induction treatment before it was possible to assess the remission status. Overall survival (OS) was defined as the time from the start of treatment to death by any cause or to the termination of observation.

### Statistical methods

Survival analysis was performed by the Kaplan-Meier method using the SPSS 13.0 software.

## Results

### Characteristics of the patients

Characteristics of the patients at the time of diagnosis are listed in Table 1. The median age of these patients was 70 (60–84) years. Seven patients had history of myelodysplastic syndrome (MDS). The median WBC count was 5.0 (0.7–263.3) × 10^9^/L, and blast cells in bone marrow was 63.0 (20.0–92.5)%. Ten patients had cytogenetics data. Among them, 2 patients had poor-risk karyotypes; 8 had intermediate-risk karyotypes. The major FAB subtypes were M4 (5/23) and M5 (7/23).

**Table 1:** Clinical data of 23 patients treated with induction chemotherapy of homoharringtonine and cytarabine.

**Case**	**Sex/year**	**History of MDS(+/-)**	**WBC (×10^**9**^)**	**BM leukemic cells(%)**	**karyotype**	**FAB subtype**
1	F/60	-	7.3	58.0	t(7;11), -21/-17	M2
2	M/70	+	2.7	31.0	-	M1
3	M/61	-	102.0	76.0	-	M5
4	F/70	-	60.4	93.0	-	M4
5	M/75	+	1.5	81.0	Normal	M6
6	M/60	+	0.7	38.0	-	M2
7	F/60	-	1.3	70.5	Normal-	M5
8	M/61	-	1.2	60.0	Normal	M5
9	M/71	-	58.9	76.0	-	M4
10	M/75	+	24.3	20.0	Normal	M4
11	M/63	-	1.3	64.0	-	M5
12	F/60	+	31.8	48.0	t(11;19)	M2
13	M/74	-	263.3	85.0	-	M4
14	M/70	-	6.2	96.0	+1(?20q-)	M1
15	M/69	-	21.7	76.0	7q-,11q-	M1
16	M/72	-	15.8	85.0	Normal	M5
17	F/70	+	1.5	20.0	-	M4
18	F/72	-	2.1	31.0	-	M7
19	F/66	-	3.0	46.0	-	M6
20	M/71	-	1.1	35.0	-	M2
21	M/84	-	11.1	72.0	Normal	M1
22	M/72	-	1.3	66.0	-	M5
23	F/70	+	4.0	35.0	-	M5

### Response

Among patients treated with HA protocol, 39.1% (9/23) achieved CR, 17.4% (4/23) achieved PR, with the overall response (OR) of 56.5% (13/23).

Three of the 7 patients who had AML secondary to MDS achieved CR and another one achieved PR. Only 1 of the 4 patients whose white blood cell count exceeded 50 × 10^9^/L at the time of diagnosis achieved CR. Two patients refused to accept consolidation therapy after CR. The rest of the patients received a median of 4 (1–12) courses of consolidation therapy after CR.

### Toxicity

The most common toxicity during the induction phase was myelosuppression. The median time from the end of chemotherapy to the neutrophil count reaching 1.5 × 10^9^/L was 16 (range: 7–45) days and the median time to the platelet reaching 50 × 10^9^/L was 15 (7–40) days in 19 patients; in the other 4 patients blood cell counts never recovered to the above level. The median duration of anti-infection (fungus or bacteria) treatment was 12 (0–46) days. The median amount of platelet transfusion was 20 (0–110) units, and red blood cell transfusion 4 (0–10) units. There was no treatment-related mortality. Non-hematological toxicities were mild, mainly nausea or emesis of I-II degree. There was no severe cardiotoxicity observed in this cohort of patients.

### Follow-up results

**Figure 1:**
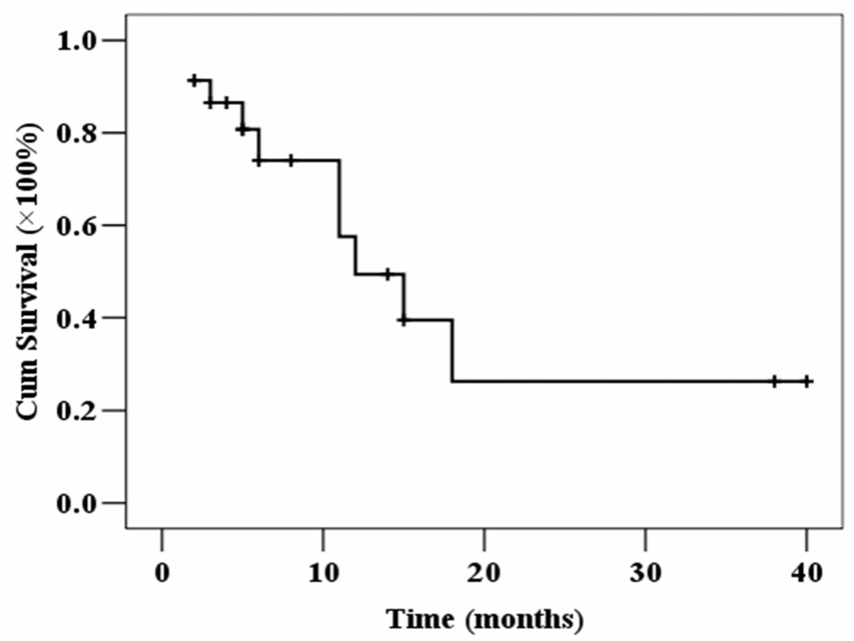
**Probability of overall survival in elderly patients with AML undergoing induction chemotherapy of homoharringtonine and cytarabine**.

The observation was terminated when the patient died, missing, or the disease free survival time reaching three years. The estimated median OS time of all patients was 12.0 ± 3.0 months. The estimated OS time of the CR patients were 15 months. The estimated one-year OS rate of all patients were 49.3 ± 13.5% (Figure 1). The estimated one-year OS rate of CR patients was 62.5 ± 17.1%.

## Discussion

Age affects survival significantly in AML patients. Elderly AML patients are generally offered palliative treatment instead of induction chemotherapy. However, studies by Pigneus and other research groups suggest that elderly patients with AML should not be excluded systematically from intensive chemotherapy protocols. They found that elderly patients who received chemotherapy achieved longer survival times than those who refused treatment or received supportive treatment alone. Unfortunately, many of these patients suffer serious or fatal complications during treatment [13-20]. For example, Alymara et al. reported a study in which 23.7% of the 38 patients older than 60 years of age who received chemotherapy using idarubicin (8 mg/m^2 ^for 3 days), Ara-c (100 mg/m^2 ^for 5 days), and etoposide (75 mg/m^2 ^for 5 days) achieved CR, while 34.2% patients achieved PR. However, during the treatment, 42.1% of their patients died of infection, cerebrovascular or gastrointestinal hemorrhage, or acute myocardial infarction [15].

An Eastern Collaborative Oncology Group study randomized elderly AML patients to remission induction therapy with either daunorubicin, idarubicin, or mitoxantrone along with a standard dose of Ara-C and priming with GM-CSF. The outcomes were not significantly different in the three arms, with CR rates ranging from 40% to 46%, median survival 8 months, and a 15% treatment related death [21].

In the present retrospective study, we have studied the outcomes in elderly patients who were treated with induction chemotherapy of HA protocol in this hospital. In the previous study of Jin et al [7], a homoharritonine-based regimen (HAA: homoharritonine 4 mg/m^2^/day, days 1–3; cytarabine 150 mg/m^2^/day, days 1–7; aclarubicin 12 mg/m^2^/day, days 1–7) was shown to be a well-tolerated, effective induction regimen in young adult patients with de novo AML. Eighty-three percent of patients achieved CR, the estimated 3 years OS rate was 53%, whereas for patients with M5, the estimated OS rate at 3 years was 75%. In our study, the response results of HA are comparable with these and other reported results in elderly patients with AML [15-21]. The response rates of HA are also comparable with the data of elderly patients treated with DA (daunorubicin 40 mg/m^2^/d for 3 days; Ara-c, 100 mg/m^2^/day for 7 days) or IDA (idarubicin 6 mg/m^2^/d for 3 days; Ara-c, 100 mg/m^2^/day for 7 days) protocols in our center during the same period. The differences in CR, OR rates and estimated median OS times between HA, DA, IDA groups were not statistically significant (Table 2). The results suggest that HA is also an effective induction regimen with less toxicity in elderly patients with AML. Furthermore, 3 of the 7 patients with AML secondary to MDS achieved CR, suggesting HA regimen is also effective in elderly patients with AML secondary to MDS. The toxicity of HA regimen protocol was relatively low. There was no early death in these patients treated with HA regimen and no severe cardiotoxicity was shown, while the ED rate within the first month of induction therapy in patients treated with DA and IDA from this same hospital was high (19.5% and 23.8%, respectively, Table 2), suggesting that HA regimen may be better tolerated in elderly patients with AML. A prospective study on this regimen for elderly AML patients is warranted.

**Table 2:** Response results of HA, DA and IDA regimens as induction chemotherapy in the treatment of elderly AML patients.

	**HA(n = 23)**	**DA(n = 21)**	**IDA(n = 21)**
CR (%)	39.1	38.1	57.1
OR (%)	56.5	47.6	61,9
ED (%)	0	19.5	23.8
OS time (m)	12.0 ± 3.0	14.0 ± 4.7	8.0 ± 1.3

HA: homoharringtonine + cytarabine; DA: daunorubicin + cytarabine; IDA: idarubicin + cytarabine.

CR: complete remission; OR: overall response; ED: early death; OS: overall survival.

## Competing interests

The authors declare that they have no competing interests.

## Authors' contributions

JW designed the research, supervised the research, analyzed the data, wrote and revised the paper. SL analyzed the data and wrote the paper; JY, XS, LC, CH, JH, WZ treated part of the patients and collected the data. All authors read and approved the final manuscript.
